# Pet distribution modelling: Untangling the invasive potential of *Trachemys dorbigni* (Emydidae) in the Americas

**DOI:** 10.1371/journal.pone.0259626

**Published:** 2021-11-11

**Authors:** Érica Fonseca, Camila Both, Sonia Zanini Cechin, Gisele Winck

**Affiliations:** 1 Departamento de Biologia, Programa de Pós-Graduação em Biodiversidade Animal, Universidade Federal de Santa Maria, Santa Maria, Rio Grande do Sul, Brazil; 2 Departamento Interdisciplinar, Universidade Federal do Rio Grande do Sul, Campus Litoral Norte, Tramandaí, Brazil; 3 Laboratoire d’Écologie Alpine (LECA), Université Grenoble Alpes, CNRS, Université Savoie Mont Blanc, Grenoble, France; Instituto Federal de Educacao Ciencia e Tecnologia Goiano - Campus Urutai, BRAZIL

## Abstract

Human activities have been changing the global biogeographic patterns by the introductions of invasive species. For reptiles, the invasion rate increase of non-native species is remarkably related to the pet trade, especially for freshwater turtles. Here we estimated the invasive potential of the South American turtle *Trachemys dorbigni* in the Americas using a combination of climatic and human activity variables. We built species distribution models based on data from the native and invasive ranges, using the ensemble model from five different algorithms (GAM, MAXENT, BRT, RF and GBM). We compared the two models’ performance and predictions, one calibrated with only climatic variables (climate-driven), and the second also included a descriptive variable of human activity (climate plus human-driven). Suitable areas for *T*. *dorbigni* covered occurrence areas of its congeners and highly diversified ecoregions, such as the eastern USA, the islands of Central America, and the south eastern and eastern Brazilian coast. Our results indicate that human activities allow *T*. *dorbigni* to establish populations outside of its original climatic niche. Including human activity variables proved fundamental to refining the results to identify more susceptible areas to invasion and to allow the efficient targeting of prevention measures. Finally, we suggested a set of actions to prevent *T*. *dorbigni* becoming a highly impacting species in the areas identified as more prone to its invasion.

## Introduction

Human activities continuously change the global biogeographic patterns through the establishment of introduction pathways of non-native species, increasing biotic exchange rate [1, 2]. As a consequence, biological invasions are among the main threats to biodiversity [3]. Increased numbers of invasive species are associated with the increasing number of individuals introduced and introduction events (the propagule pressure; [4, 5]). Biological invasion theory deals with the invasion process as a multistage continuum, where a high propagule pressure is essential for the successful establishment and maintenance of small populations during the early stages of invasion (sensu [6]). This high propagule pressure allows a continuous gene flow even in nonsuitable areas and, therefore, increases the probability of adaptation [7]. Specifically in the pet trade, the continuous release of individuals into nature reduces the reliance on reproductive success for long-term population maintenance, which increases the chances of establishment. The number of invasive species introduced by the legal and illegal pet trade has increased over the last decades worldwide, influenced by commercial expansion through online commerce, and especially in social networks [8–10]. New invasions are expected to continually emerge in the next years due to a lag phase, when species remain with few individuals in the introduction area for a long time before becoming invasive [11, 12]. Therefore, initiatives to identify the most prone areas to invasion before these nonindigenous species become invasive are critical for conserving native populations and planning for the prevention and management of invasive species.

Both biotic (e.g., competition, facilitation, vegetation cover) and abiotic (e.g., temperature, humidity) filters also influence the successful establishment of an invader species [13]. These environmental constraints are often associated with physiological limitations and demands for suitable sites for reproduction. Despite the general lack of detailed ecological data for most invasive populations, a general macroecological approach using spatial statistical models can provide important information from the available biotic and abiotic data (e.g., [14–17]. Species distribution modelling (SDM) is a commonly used tool to estimate suitable areas for nonindigenous species. SDMs assist in risk assessment by identifying areas prone to invasions and provides valuable information for mitigation measures ([18, 19]. These models can be used in a more comprehensive approach by including other environmental variables, such as vegetation cover, invasive species introduction events, or transport pressure (see [15]). For invasive species modelling, the inclusion of variables that quantify human activity in SDM improves model estimation power [20, 21]. For example, exotic pet introduction depends on their release into the environment; thus, propagule pressure for these animals should be higher in large urban centres and their surroundings, and closer to residences [22, 23]. As a result, the inclusion of human activity measures in the SDM allows combining the probability of introduction or release (and accessibility) with climatic suitability of habitat [24, 25] to provide more realistic scenarios.

In this study, we used SDMs to understand the invasive risk of a South American freshwater turtle, *Trachemys dorbigni* (Duméril and Bibron, 1835), within the Americas. This species is native to southern Brazil, Uruguay and northern Argentina [26, 27], inhabiting different aquatic environments with abundant vegetation (lakes, slow-flowing rivers, ponds, and wetlands), and urban areas [28, 29]. Due to pet release, *T*. *dorbigni* was introduced in areas outside of its native distribution, becoming invasive at some locations in north-eastern and south-eastern Brazil. However, its population impacts are still poorly known [30, 31]. This study is the first approach on its potential for invasion, providing information to prevent future impacts on potential invaded communities and freshwater ecosystems, since one of its congener (*Trachemys scripta*; Thunberg in Schoepff, 1792) figures as a top-100 worst invasive species of the world. Banning *T*. *scripta* trade in Europe and in some South American countries [32–34] increased pressure upon other species of freshwater turtles [34], including our targeted species. Indeed, egg and new-born removal for the pet trade is the main threat within its native area, together with water pollution and road kills [35]. Here we combine two sets of variables to model habitat suitability for *Trachemys dorbigni* in the Americas, one including only climatic (abiotic) variables and the second including a human activity index; this allowed us to: (1) identify which areas are more prone to *T*. *dorbigni* invasion, and (2) to analyse human activity as a major driver of increased environmental suitability for this species.

## Methods

### Species occurrence data

We compiled occurrence records of *Trachemys dorbigni* in its native range and invaded areas from the global biodiversity information facility (GBIF; [36]), the VertNet data portal (www.vertnet.org), Hórus Institute [37], and peer-reviewed published articles (S1 Appendix). Our data set included the largest number of occurrence records with a minimal pairwise distance of 10 Km to prevent sampling bias [38], using the randomisation function from ‘spThin’ R package [39]. The final dataset contained 56 occurrence records of *T*. *dorbigni* within its native range, plus 15 invasive population records. Because the species is freshwater-dependent, presence records are geographically biased towards rivers and lakes.

### Climatic variables and measures of human activity

We included the 19 bioclimatic variables from CHELSA [40] and two topographic variables (elevation and slope) derived from the Hydro-1K global digital elevation model [41]. Since we were explicitly interested in how human activity influences the invasion potential of *T*. *dorbigni*, we included the global human influence index (HII; [42]) as a second data set, containing bioclimatic, topographic, and human influence variables. HII is built up from nine global data layers including three main anthropogenic pressure sources [42], human population (density), land use and infrastructure (e.g., land cover), and human accessibility (e.g., roads). All layers had a spatial resolution of 5 arc-minutes (approximately 10 Km x 10 Km at the equator).

To prevent multicollinearity, we selected the two sets of variables with the lowest correlation values based on the variance inflation factor (VIF) using the ‘fmsb’ R package [43]. VIF is an effective approach for multicollinearity assessment for more than two independent variables at a time; the higher the VIF value, the higher the collinearity between the related variables [44]. We only selected the bioclimatic variables below the critical threshold of five: isothermality (bio 3), temperature annual range (bio 7), mean temperature of the wettest quarter (bio 8), mean temperature of the warmest quarter (bio 10), and precipitation seasonality (bio 15). Therefore, one predictor dataset comprised only the climatic variables selected by VIF, and other combined the climatic variables and the human influence index.

### Species distribution models

We calibrated two SDM sets for *T*. *dorbigni*: one set using only climate variables (climate-driven; CD) with both native and invasive occurences, and another set including human-related variables (climate plus human-driven; CHD), using only invasive occurrences. To assess the role of anthropogenic activities on the environmental suitability and the invasive potential of *T*. *dorbigni*, we jointly used native and invasive occurrences. We believe joining occurrences would improve its fundamental niche characterization, comparing to models with native occurrences only [18]. Likewise, we run CHD models with invaded occurrences only to incorporate anthropogenic effects on the release and/or escape of exotic species. Human activity may produce an opposite effect on the native area, negatively impacting the species instead of allow its expansion [45, 46]. We projected the mean resulting models (CD and CHD models) into wider-ranged layers (the Americas) to identify the most suitable areas for population establishment. We assumed that higher-valued suitability areas were more prone to successful invasions.

We identified the areas with higher invasive potential for the turtle using the ensemble model from five different algorithms (GAM, MAXENT, BRT, RF and GBM) through the ‘biomod2’ R package [47]. Ensemble models can provide more robust predictions for potential distributions of invasive species, where species-environment relationships are difficult to determine [48, 49]. Ensemble distribution maps highlight areas of agreement among different models predictions, reducing areas of uncertainty for individual models [50, 51].Because these algorithms require presence and absence records, and absence records are usually either unavailable or unreliable, we replaced them by pseudo-absence occurrence sets [52]. We generated a random sample set of pseudo-absences proportional to the number of presences [53] with three replicates within the study area. We set equal prevalence weight for both presence and pseudo-absence groups (0.5; [54]) to prevent model overfitting and to produce a more accurate prediction for potential invasions [53, 55–57].

For model evaluation, we randomly divided the data set into 80% for calibration and 20% for validation. We repeated this procedure 10 times using 10 random data set splits, totalling 100 models for each type of model (CD and CHD). To evaluate model-fitting, we used the true skill statistic (TSS). TSS is a precision-dependent measure, insensitive to prevalence [58]. To build a consensus prediction, we only included higher-performance models in the ensemble forecasting (TSS >0.7; [59]). We compared the TSS values of the CD and CHD models using Wilcoxon’s statistics to verify if the different sets of variables affected the performance of the models. Final outputs were two continuous suitability layers comprising the mean ensemble of the best predictions for each model type (CD and CHD), both projected in the Americas. Continuous predictions were converted into binary maps by using a threshold value which maximized both sensitivity and specificity to show the differences and convergences between each model.Unlike most studies with invasive species, here we prioritised maximising specificity (the proportion of absences correctly predicted) in defining model parameters; that is, we chose predictions with higher specificities to reduce the number of false positives (true absences predicted as presence; [53]). The ensemble distribution maps represent only the areas with the greatest potential to be suitable for *T*.*dorbigni*.

## Results

**Fig 1:**
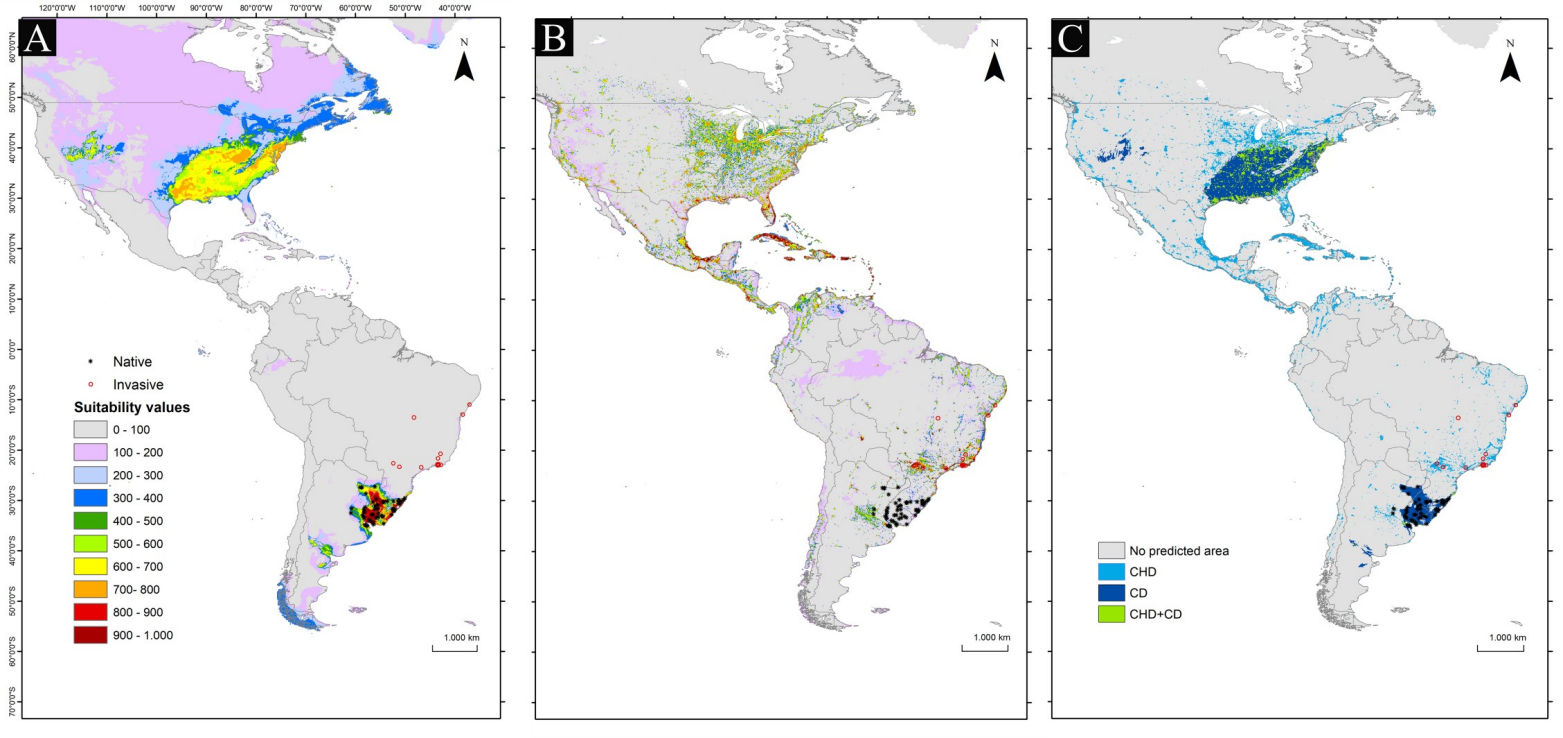
The resulting suitability areas for the successful establishment of *T*. *dorbigni* in the Americas. (**A**) climate-only model (CD); (**B**): climate + human activity model (CHD); (**C**) comparison of predicted distributions in the CD and CHD models; light blue areas show where the CHD model predicts species presence, while dark blue areas areas show where the CD model predicts species presence. Areas in green indicate coincidence in model predictions. Map images hosting provided by the Center for Spatial Sciences at the university of California, Davis.

As expected, in the climate plus human-driven model (CHD) suitable areas coincided with areas in which the *Trachemys dorbigni* is invasive (Fig 1). Using the ensemble approach, the climate-driven model (CD) identified suitable areas for *T*. *dorbigni* in north-central Argentina along the Rio Negro drainage basin, eastern USA, and the Great Basin in the USA (sensu [60]), in addition to their natural range (Fig 1A). These ecoregions are typically arid and temperate, presenting independent river systems and drainage basins [61, 62]. Other smaller areas of dry and moist forests in the south-eastern and eastern Brazilian coast, northern Andean region, Caribbean islands, Central America, and parts of Chile were identified by the CHD model (Fig 1B). A comparison between the distributions predicted by both models (CD and CHD) can be found in Fig 1C.

**Fig 2:**
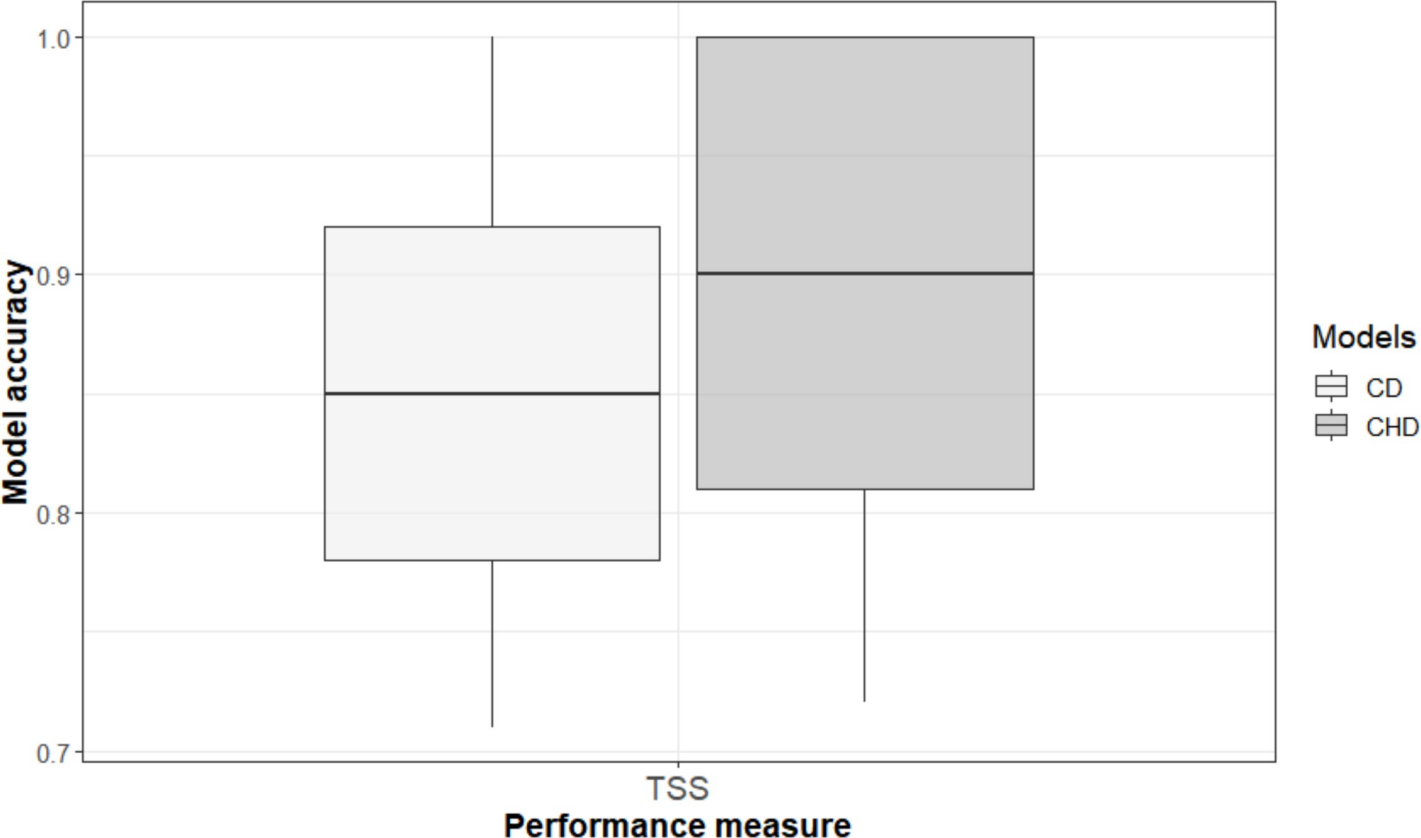
TSS values for the climate-driven model and combined model (climate and human activity index).

TSS values for ensemble models are 0.87 for CD model and 0.99 for CHD model, which is considered a good performance (Fig 2). However, the CHD model differed significantly from CD, with higher TSS (Wilcoxon rank-sum test on TSS (W = 16145, *p* = 2.895e-11). Therefore, our results from both models comprised good predictions on habitat suitability for the species, but the CHD model performed better according to model evaluation metrics.

## Discussion

The CHD model outperformed the CD model at predicting known invaded areas, and in model precision. This suggests that human-driven environmental modifications allow *T*. *dorbigni* to establish populations outside its original climatic niche. Although the successful establishment is influenced by propagule pressure and abiotic and biotic characteristics of the invaded area, the magnitude and extent of the effect of each factor are often modified by human activity [13]. Human activity increases the likelihood of population establishment, by increasing genetic diversity and the chances of adaptation through the constant release of pets (see [7]). At the same time, environmental modification creates favourable environments (e.g., increased local temperature, lack of vegetation cover) and facilitates the occupation of empty niches by non-native species [63, 64]. Therefore, if human activity at the site is high enough, it may favor the establishment of exotic species in areas outside the appropriate climatic range. Population establishment of invasive species in unfavourable climatic regions is not an uncommon event; for example, introduced populations of *T*. *scripta* in Italy usually survive in habitats where bioclimatic conditions are unfavourable to reproduction [65]. This may be the case for populations of *T*. *dorbigni* inhabiting areas outside of their suitable range, since most of their invasion sites are deforested areas within the Brazilian Atlantic Forest domain, where invasion risks and propagule pressure are higher, due to the influence of the 72% (ca. 145 million) of the human population Brazilian living within its domain [66]. Almost all of the sites invaded by *T*. *dorbigni* are located in the Atlantic Forest region and were all correctly predicted by the CHD model.

Suitable areas for *T*. *dorbigni* also agreed with the distribution of *T*. *scripta* in North America, as well as the distribution of other slider turtles (*Trachemys* sp.) in Central and South America, especially *T*. *stejnegeri*, *T*. *decussata*, *T*. *terrapen and T*. *venusta* (S1 Fig). These results suggest that *Trachemys* species occupy similar habitats and share characteristics of life history [67]; this is especially worrying for the maintenance of native congener populations, mainly because non-native reptiles are overall more likely to successfully establish where congeners are present [67]. According to Darwin’s preadaptation hypothesis, the presence of congeners indicates that resource availability and physiological tolerances are compatible with local conditions [68]. However, the relationship between congener presence and the successful invasion of turtles on a global scale has not yet been demonstrated [69]. Further analyses on a smaller scale (regional and local) may clarify the importance of congeners in the success of invasive slider turtles. In contrast, the impacts of introducing non-native slider turtles are known and include changes in behaviour and distribution of native turtle populations by biotic interactions, disease spread, and hybridisation [70, 71]. Hybridisation cases involving *Trachemys* species are already observed [71, 72] suggesting the lack of physiological barriers between species. In a successful invasion scenario of *T*. *dorbigni* in congener areas, the phylogenetic proximity between *Trachemys* species suggests that their hybridisation could occur [73, 74]. In addition, sliders are considered highly competitive and aggressive turtles in their interactions [75, 76]. Competition for food, and nesting and basking sites displaces native turtle species from the most suitable places for thermoregulation and where resources are most profitable [75, 77]. Competitive displacement, together with hybridisation, can alter population structure and endanger the survival of native populations, decreasing reproductive success, and increasing mortality rates [70, 78].

Given the stochastic nature of each invasion and the uncertainties related to the introduction of new propagules, to precisely predict which areas are most at risk becomes a challenge. Although climatic variables are important, especially for ectothermic species, they alone are not enough to describe invasive species distribution [79]. The occurrence of *T*. *dorbigni* can be also limited by other factors besides thermal tolerance [80]. As freshwater invaders, these organisms are more prone to occupy large areas by using water body networks for dispersal, together with the known pet introduction pathways. The accessibility to other lakes, ponds, and rivers may be key for the spread of this species, and difficulties for dispersal process include habitats split by dams and roads, for example. In this sense, alternative models run with the freshwater environmental database and other variables associated with the presence of water can refine future models and improve predictions by including due inclusion of likely dispersal routes for aquatic invaders [45]. Furthermore, it is likely that its occurrence is also limited by density-dependent factors (e.g., intraspecific competition, density, mortality rates) which reduces the need of individuals to disperse. Therefore, local abiotic and biotic patterns also exert pressure on the establishment and dispersion of *T*. *dorbigni*, and should be considered in future plans to contain and prevent invasion of the species.

Here we used species distribution models not only to identify areas more prone to *T*. *dorbigni* invasion, and which variables would explain better the suitability, but also to understand the role of human activities on the invasion process. Our results comply with previous studies highlighting the importance of including human activities as variables in SDMs to improve model predictions of invasive species, especially freshwater turtles [65, 79, 81, 82]. Because human activity is highly influential in the invasion process, we believe that even when calibrating models that account for the interaction between species (e.g., JSDM, Joint Species Distribution Model), the human variable should enhance the prediction power of invasion-prone areas. However, it is important to highlight that the SDMs consider that the species would be able to reach all areas equally and that the conditions will remain the same offered in the construction of the model. Thus, changes in parameters (e.g., changes in accessibility or vegetation cover) may affect these projections and, therefore, the interpretation of these results must consider such limitations.

In a nutshell, *T*. *dorbigni* invasiveness is driven by both climatic features and human activities. Our study generated important information to employ in direct conservation efforts at specific geographic areas in the Americas, since the habitat suitability for *T*. *dorbigni* includes the occurrence areas of its congeners and highly diverse ecoregions with great importance for conservation. Therefore, we believe the most imperative actions to prevent *T*. *dorbigni* from becoming a highly impacting non-native species within prone-invasive areas are: 1) limit or even ban commercial trade. Alternatively, all breeders and shops must implant a subcutaneous microchip for identification of the turtle, and the commercial establishment should register the owner in a country and/or worldwide database of non-native species. 2) Authorities and governments to set high fines for those who release individuals, and establish inspection activity on pet breeders and shops. 3) To promote citizen science programs to increase invasive species monitoring areas, including a user-friendly database to report findings and data. The threat of invasive species can only be mitigated by the close cooperation of the triad of scientists, civil society, and policymakers.

## Supporting information

S1 Fig Native distribution of the *Trachemys* genus in the Americas. Black polygon: geographical distribution of *T*. *dorbigni*. Red polygons:1- *T*. *scripta*; 2- *T*. *venusta*; 3- *T*. *decussata*; 4- *T*. *gaigeae*; 5- *T*. *grayi*; 6- *T*. *nebulosa*; 7- *T*. *ornata*; 8- *T*. *stejnegeri*; 9- *T*. *taylori*; 10- *T*. *terrapen*; 11- *T*. *yaquia*; 12- *T*. *adiutrix*. Modified from Fritz (2012). Map images hosting provided by the Center for Spatial Sciences at the university of California, Davis. CD: climate-only model, CHD: climate + human activity model.
(DOCX)

S1 Table Geographic coordinates, states and country of occurrence records of *Trachemys dorbigni*. (DOCX)

S2 Table Variables importance (mean values ± SD) from the climate-only (CD) and combined (climate + human activity; CHD) models for *Trachemys dorbigni*. Variables: bio3 = isothermality; bio7 = temperature annual range; bio8 = mean temperature of wettest quarter; bio10 = mean temperature of warmest quarter; bio15 = precipitation seasonality; HII = human influence index.
(DOCX)

S1 Appendix List of published papers holding *Trachemys dorbigni* occurrence information. (DOCX)
