# Supplementary material for: Pet distribution modelling: Untangling the invasive potential of Trachemys dorbigni (Emydidae) in the Americas
Source: PLoS One. 2021 Nov 11;16(11):e0259626. doi: 10.1371/journal.pone.0259626 (PMC8584657; doi:10.1371/journal.pone.0259626)

**S1 Fig. Native distribution of the *Trachemys* genus in the Americas**. Black cicle: geographical distribution of *T. dorbigni.* Red polygons:1- *T. scripta*; 2- *T. venusta*; 3- *T. decussata*; 4- *T. gaigeae*; 5- *T. grayi*; 6- *T. nebulosa*; 7- *T. ornata*; 8- *T. stejnegeri*; 9- *T. taylori*; 10- *T. terrapen*; 11- *T. yaquia*; 12- *T. adiutrix*. Modified from Fritz (2012). Map images hosting provided by the Center for Spatial Sciences at the university of California, Davis. CD: climate-only model, CHD: climate + human activity model.


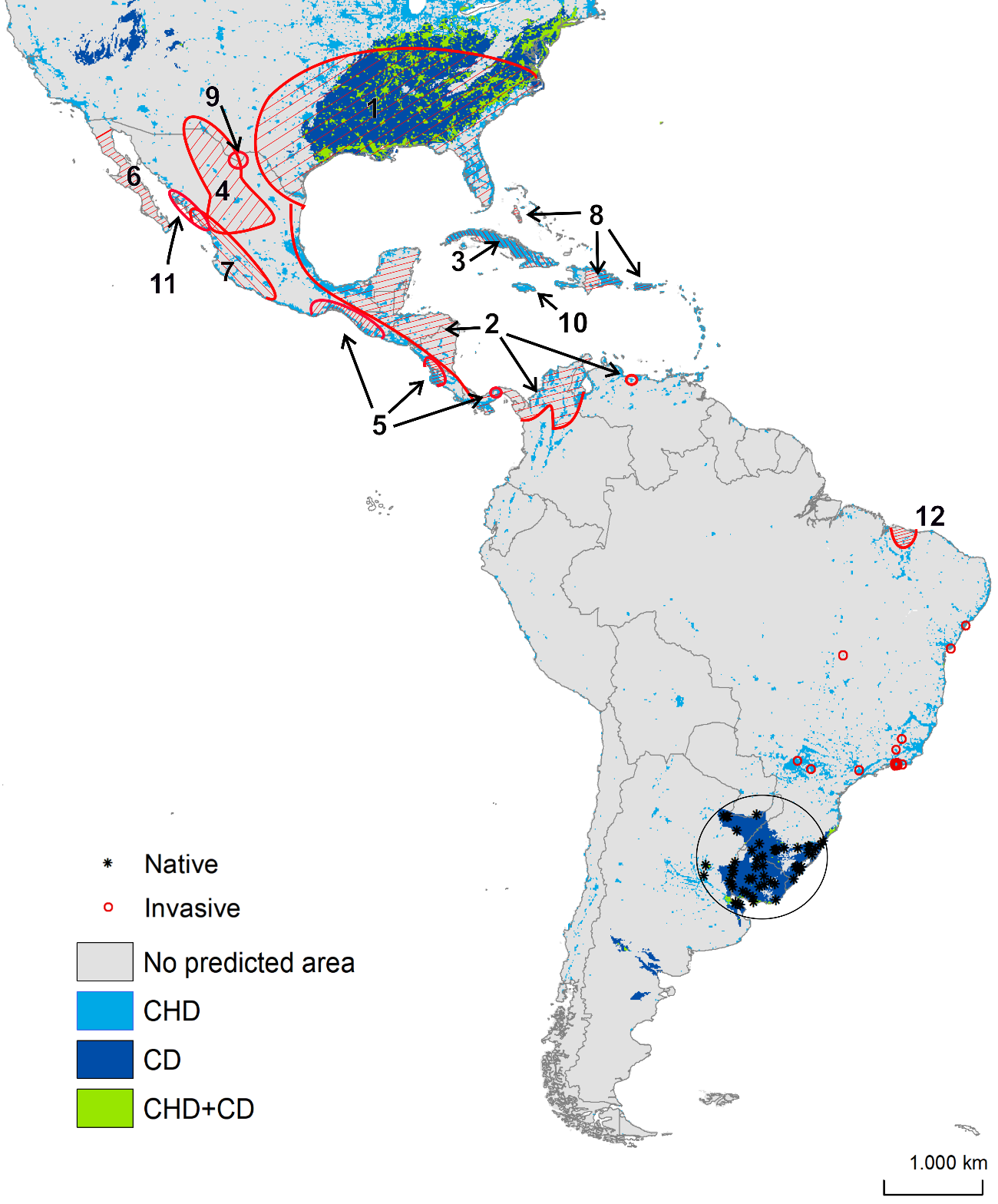

Supplement: S1 Fig — Black polygon: geographical distribution of T. dorbigni. Red polygons:1- T. scripta; 2- T. venusta; 3- T. decussata; 4- T. gaigeae; 5- T. grayi; 6- T. nebulosa; 7- T. ornata; 8- T. stejnegeri; 9- T. taylori; 10- T. terrapen; 11- T. yaquia; 12- T. adiutrix. Modified from Fritz (2012). Map images hosting provided by the Center for Spatial Sciences at the university of California, Davis. CD: climate-only model, CHD: climate + human activity model. (DOCX) [file pone.0259626.s001.docx]
