# Supplementary material for: Pet distribution modelling: Untangling the invasive potential of Trachemys dorbigni (Emydidae) in the Americas
Source: PLoS One. 2021 Nov 11;16(11):e0259626. doi: 10.1371/journal.pone.0259626 (PMC8584657; doi:10.1371/journal.pone.0259626)
Supplement: S1 Table — (DOCX) [file pone.0259626.s002.docx]

**S1 Table. Geographic coordinates, states and country of occurrence records of *Trachemys dorbigni*.**

| Longitude | Latitude | Country | Locality | Status |
| --- | --- | --- | --- | --- |
| -60.837263 | -32.560136 | Argentina | Santa Fe | Native |
| -60.697294 | -31.610658 | Argentina | Santa Fe | Native |
| -59.053374 | -27.405438 | Argentina | Chaco | Native |
| -58.783346 | -27.452093 | Argentina | Corrientes | Native |
| -58.658636 | -27.456495 | Argentina | Corrientes | Native |
| -58.52486 | -33.01215 | Argentina | Entre Rios | Native |
| -58.311535 | -33.28912 | Uruguay | Fray Bentos | Native |
| -58.26161 | -31.88425 | Argentina | Entre Rios | Native |
| -58.24741 | -32.47959 | Uruguay | Concepción Del Uruguay | Native |
| -58.221876 | -33.518501 | Uruguay | Soriano | Native |
| -58.1333 | -32.15 | Uruguay | Paysandu | Native |
| -58.02178 | -31.37743 | Argentina | Concordia | Native |
| -57.965592 | -34.825534 | Argentina | Punta Lara | Native |
| -57.953566 | -34.920495 | Argentina | Buenos Aires | Native |
| -57.85809 | -28.68172 | Argentina | Corrientes | Native |
| -57.7 | -34.983333 | Argentina | Arroyo Zapata | Native |
| -57.543818 | -35.04044 | Argentina | Buenos Aires | Native |
| -57.300512 | -33.884523 | Uruguay | Soriano | Native |
| -56.915047 | -34.053623 | Uruguay | São Jose | Native |
| -56.765899 | -32.841115 | Uruguay | Soriano | Native |
| -56.470683 | -30.387693 | Brazil | Rio Grande do Sul | Native |
| -56.390423 | -34.398787 | Uruguay | Florida | Native |
| -56.367349 | -32.800856 | Uruguay | Lago de Ricon | Native |
| -56.358333 | -34.786111 | Uruguay | Montevideo | Native |
| -56.125099 | -31.813269 | Uruguay | Los Rosanos | Native |
| -56.030972 | -27.274741 | Argentina | Rio Paraná | Native |
| -56.014906 | -31.145241 | Uruguay | Rivera | Native |
| -55.81 | -33.5 | Uruguay | Florida | Native |
| -55.780762 | -29.779721 | Brazil | Rio Grande do Sul | Native |
| -55.522656 | -30.866526 | Brazil | Rio Grande do Sul | Native |
| -55.506718 | -30.933209 | Uruguay | Rivera | Native |
| -55.460640 | -31.660365 | Uruguay |  | Native |
| -55.37 | -32.6 | Uruguay | Arroyo del Estado | Native |
| -55.339690 | -33.347344 | Uruguay | Florida | Native |
| -54.768005 | -33.042410 | Uruguay | Treinta y Tres | Native |
| -54.411111 | -30.313611 | Brazil | Rio Grande do Sul | Native |
| -54.367313 | -33.273021 | Uruguay | Treinta y Tres | Native |
| -54.305899 | -30.334391 | Brazil | Rio Grande do Sul | Native |
| -54.297075 | -34.627306 | Uruguay | La Paloma | Native |
| -54.261389 | -30.341667 | Brazil | Rio Grande do Sul | Native |
| -54.259088 | -30.281919 | Brazil | Rio Grande do Sul | Native |
| -53.552098 | -30.164355 | Brazil | Rio Grande do Sul | Native |
| -52.643856 | -32.834264 | Brazil | Rio Grande do Sul | Native |
| -52.513333 | -31.800278 | Brazil | Rio Grande do Sul | Native |
| -52.340278 | -31.77 | Brazil | Rio Grande do Sul | Native |
| -52.293899 | -30.166674 | Brazil | Rio Grande do Sul | Native |
| -52.179286 | -32.132615 | Brazil | Rio Grande do Sul | Native |
| -52.106066 | -32.035585 | Brazil | Rio Grande do Sul | Native |
| -51.943806 | -31.790989 | Brazil | Rio Grande do Sul | Native |
| -51.345397 | -30.566509 | Brazil | Rio Grande do Sul | Native |
| -51.316111 | -29.983056 | Brazil | Rio Grande do Sul | Native |
| -51.18 | -30.050556 | Brazil | Rio Grande do Sul | Native |
| -51 | -30.766667 | Brazil | Rio Grande do Sul | Native |
| -50.682786 | -30.229976 | Brazil | Rio Grande do Sul | Native |
| -50.211878 | -29.887084 | Brazil | Rio Grande do Sul | Native |
| -49.995428 | -29.574376 | Brazil | Rio Grande do Sul | Native |
| -38.41135 | -12.968436 | Brazil | Bahia | Invasive |
| -48.22156 | -13.527858 | Brazil | Goiás | Invasive |
| -43.40291 | -21.683936 | Brazil | Minas Gerais | Invasive |
| -43.49872 | -22.83855 | Brazil | Rio de Janeiro | Invasive |
