# Supplementary material for: Pet distribution modelling: Untangling the invasive potential of Trachemys dorbigni (Emydidae) in the Americas
Source: PLoS One. 2021 Nov 11;16(11):e0259626. doi: 10.1371/journal.pone.0259626 (PMC8584657; doi:10.1371/journal.pone.0259626)
Supplement: S2 Table — Variables: bio3 = isothermality; bio7 = temperature annual range; bio8 = mean temperature of wettest quarter; bio10 = mean temperature of warmest quarter; bio15 = precipitation seasonality; HII = human influence index. (DOCX) [file pone.0259626.s003.docx]

**S2 Table.** **Variables importance (mean values ± SD) from the climate-only (CD) and combined (climate + human activity; CHD) models for *Trachemys dorbigni.*** Variables: bio3 = isothermality; bio7 = temperature annual range; bio8 = mean temperature of wettest quarter; bio10 = mean temperature of warmest quarter; bio15 = precipitation seasonality; HII = human influence index. Numbers in bold highlight the variables that most contribute to each model.

|  | **Models** | |
| --- | --- | --- |
| **Variables** | Climate only | Climate + Human activity (HII) |
| Bio3 | **0.32 ± 0.22** | 0.21 ± 0.26 |
| Bio7 | **0.27 ± 0.24** | 0.12 ± 0.18 |
| Bio8 | 0.24 ± 0.19 | **0.24 ± 0.30** |
| Bio10 | 0.16 ± 0.17 | **0.29 ± 0.29** |
| Bio15 | **0.37 ± 0.24** | 0.20 ± 0.29 |
| HII | – | **0.24 ± 0.26** |
