# Supplementary material for: Pet distribution modelling: Untangling the invasive potential of Trachemys dorbigni (Emydidae) in the Americas
Source: PLoS One. 2021 Nov 11;16(11):e0259626. doi: 10.1371/journal.pone.0259626 (PMC8584657; doi:10.1371/journal.pone.0259626)
Supplement: S1 Appendix — (DOCX) [file pone.0259626.s004.docx]

**S1 Appendix. List of published papers holding *Trachemys dorbigni* occurrence information*.***

Alcalde, L.; Derocco, N.N.; Rosset, S.D. & Williams, J.D. (2012). Southernmost localities of *Trachemys dorbigni* and first record of *Trachemys scripta elegans* for Argentina (Cryptodira: Emydidae). Chelonian Conservation and Biology, 11(1): 128- 133.

Bager, A.; de Freitas, T.R.O. & Krause, L. (2007). Nesting ecology of a population of *Trachemys dorbignyi* (Emydidae) in southern Brazil. Herpetologica, 63: 56–65.

Bager, A.; Krause, L. & de Freitas, T.R.O. (2012). Fidelity to nesting sites and orientation of *Trachemys dorbigni* (Duméril & Bibron, 1835) (Testudines: Emydidae) female in southern Brazil, Tropical Zoology, 25(1): 31-38. DOI: 10.1080/03946975.2012.679393

Bernardon, F.F.; Valente, A.L. & Müller, G. (2014). Gastrointestinal helminths of *Trachemys dorbigni* Duméril & Bibron, 1835 (Testudines, Emydidae) from artificial urban ponds in southern Brazil. Pan-American Journal of Aquatic Sciences, 9(1):54-57.

Borges-Martins, M. et al. (2007). Répteis, In: Becker, F.G.; Ramos, R.A.; Moura, L. A. (Orgs.) Biodiversidade: Regiões da Lagoa do Casamento e dos Butiazais de Tapes, Planície Costeira do Rio Grande do Sul. Ministério do Meio Ambiente, Brasília, p. 292-314.

Bujes, C.S. (2010). Os Testudines continentais do Rio Grande do Sul, Brazil: taxonomia, história natural e conservação. Iheringia, Série Zoologia, 100: 413–424.

Bujes, C.S. & Verrastro, L. (2008). Quelônios do delta do Rio Jacuí, RS, Brazil: uso de hábitats e conservação. Natureza & Conservação, 6: 47–60.

Coelho, I. P.; Kindel, A.; Coelho, A. V. P. (2008). Roadkills of vertebrate species on two

highways through the Atlantic Forest Biosphere. Eur J Wildl Res, 54:689–699

Cunha, G.G.; Hartmann, M.T.; Hartmann, P.A. (2015). Atropelamentos de vertebrados em uma área de Pampa no sul do Brazil. Ambiência Guarapuava (PR), 11(2): 307 – 320.

Global Biodiversity Information Facility 2016. GBIF Data Portal.

Hengemuhle, A.; Cademartori, C. V. (2008). Levantamento de mortes de vertebrados silvestres devido a atropelamento em um trecho da estrada do mar (RS-389). Biodiversidade Pampeana, 6 (2), p. 4-10.

Instituto Horus (2018). Retrieved April , 2018. <http://i3n.institutohorus.org.br/www/?p=Z2tiIX83ajs6NWgpKk1WHEpYCVwCUkBPSB5cOz47LWxrbQ%3D%3D>

Mascarenhas, C.S. & Müller, G. (2015). Third-stage larvae of the enoplid nematode *Dioctophyme renale* (Goeze, 1782) in the freshwater turtle *Trachemys dorbigni* from southern Brazil. Journal of Helminthology, 89: 630–635.

Pazinato, D.M.M.; Silva, D.E.; Corrêa, L.L.C. & Capellari, L.H. (2013). Diversidade de répteis em uma área da região centra do Rio Grande do Sul. Perspectiva. 37(137): 115-122.

Rocha, D.F.N. de B. (2005). Biologia termal das tartarugas *Trachemys dorbigni* (Duméril & Bibron, 1835) e *Trachemys scripta elegans* (Wied, 1893), dos lagos de Porto Alegre, RS, Brazil (Testudines, Emydidae). Dissertação de mestrado, Universidade Federal do Rio Grande do Sul, Rio Grande do Sul, 79 pp.

Santos TG, Vasconcelos TS, Molina FB, Zaher H (2009) First record of *Trachemys dorbigni* (Duméril & Bibron, 1835) (Testudines, Emydidae) in a remnant of Mesophytic Semideciduous Forest of Sao Paulo State, southeastern Brazil. Herpetological Bulletin 108: 27-30.

Silveira, M.L.; Hartmann, M.T.; Bager, A. Biometria, razão sexual e dimorfismo sexual de *Trachemys dorbigni* (Duméril & Bibron 1835) (Testudines, Emydidae) em um açude no município de São Gabriel, Rio Grande do Sul, Brazil. Biotemas, 25(3): 187-193.

Silveira, M.L. (2013). Variação morfológica e populacional de *Trachemys dorbigni* (Testudines, Emydidae) no extreme sul do Brazil. Dissertação de mestrado. Universidade Federal de Lavras, Minas Gerais. 91pp.

Souza-Filho G.A. & L. Verrastro. (2012). Reptiles of the Parque Estadual de Itapuã, state of Rio Grande do Sul, southern Brazil. Check List, 8(5): 847–851. http://www.checklist.org.br/getpdf?SL054-12

Steil, L.; Düpont, A.; Lobo, E.A. (2016). Levantamento da fauna silvestre atropelada na BR 290 (km 210 a 214), Município de Pantano Grande, RS, Brazil. Caderno de Pesquisa, série Biologia, 28(1): 13-23.

Quintela, F.M.; Pinheiro, R. M. & Loebmann, D. (2011). Composição e uso do habitat pela herpetofauna em uma área de mata paludosa da Planície Costeira do Rio Grande do Sul, extremo sul do Brazil. Revista Brazileira de Biociências, 9: 6–11.

VertNet 2016. Global museum database Portal.
